# Fine-Scale Vegetation Characteristics Drive Insect Ensemble Structures in a Desert Ecosystem: The Tenebrionid Beetles (Coleoptera: Tenebrionidae) Inhabiting the Ulan Buh Desert (Inner Mongolia, China)

**DOI:** 10.3390/insects11070410

**Published:** 2020-07-02

**Authors:** Yiping Niu, Guodong Ren, Giulia Lin, Letizia Di Biase, Simone Fattorini

**Affiliations:** 1 Institute of Life Sciences and Green Development, College of Life Sciences, Hebei University, Baoding 071002, Hebei, China; niu_20040118@126.com; 2 Museum of Hebei University, Baoding 071002, Hebei, China; 3 Via Principe Amedeo 175, 00185 Rome, Italy; giulia.lin997@gmail.com; 4 Department of Life, Health and Environmental Sciences, University of L’Aquila, Via Vetoio, Coppito, 67100 L’Aquila, Italy; letizia.dibiase@graduate.univaq.it

**Keywords:** arid ecosystems, Asia, community ecology, diversity, Gobi Desert

## Abstract

In community ecology, ensembles are defined as phylogenetically bounded groups of species that use a similar set of resources within a community. Tenebrionids are a conspicuous faunal component of Asian deserts, but little is known about their community ecology. We investigated if tenebrionids associated with different plant species constitute ensembles with a different ecological structure. Sampling was done with pitfall traps placed beneath the most common plant species. Tenebrionid abundance patterns were modelled by fitting rank–abundance plots. The association between tenebrionid species and plant species was tested using contingency tables. Differences in ensemble diversity were investigated by diversity profiles. All ensembles were fitted by the geometric series model. Tenebrionid species were differently associated with different plant species. Diversity profiles indicate that different ensembles have different diversity patterns, because of differences in species relative abundance. Tenebrionids form different ensembles associated with the different dominant plant species. All these ensembles are, however, characterized by similar patterns of dominance, following the “niche pre-emption” model, and a steep decline in the diversity profiles. This indicates that similar environmental conditions lead to similar insect ensemble organization, although the most abundant species may vary, which suggests a role for microhabitat selection.

## 1. Introduction

In ecology, the word community is typically used to indicate a group of interacting species occurring together in space, without reference about their possible phylogenetic relationships and resource use [1,2]. Groups of species utilizing a shared resource (but without regard to their phylogenetical relationships) are indicated as guilds, whereas phylogenetically bounded groups of species that use a similar set of resources within a community form “ensembles” [3,4,5,6]. Most research has been addressed to investigate insect communities, whereas guilds have rarely been studied, because of the lack of ecological information on resources use [7,8,9,10]. The study of insect ensembles seems to be an even more difficult task because this implies that species (i) belong to the same monophyletic group, (ii) are synchronically present in the same place, and (iii) exploit the same class of environmental resources in a similar way. Thus, research on insect ensembles is still very limited, in comparison with community and guild studies [4,5,11].

Desert ecosystems may represent ideal models to investigate the structure of insect ensembles because of various reasons. First, deserts host insect communities typically represented by relatively few species, thus surpassing the so called “taxonomic impediment” [12]. Second, in desert ecosystems, resources are scarce, and their variety is reduced, which forces most species to use the same resources, which is a prerequisite to assume they belong to the same ensemble. Third, due to the extremely high temperatures recorded during the day, especially on the soil in certain seasons, most of the ground dwelling species are active for a limited number of hours depending on the season, but typically from the sunset to the sunrise, so that their activity rhythms largely overlap, and hence they are forced to interact [13,14,15,16,17].

Among the few animal groups that have radiated in arid environments, the tenebrionid beetles (Coleoptera Tenebrionidae) represent probably the most diversified and abundant taxon in desert ecosystems worldwide [18,19,20]. Thus, several aspects make desert tenebrionids excellent model organisms for the study of ensemble ecology. First, tenebrionids are abundant and fairly easy to sample by pitfall traps, which makes it possible to obtain sufficiently large samples of individuals in a short time and even from very small sampling areas [21,22,23,24], thus ensuring that the sampled species belong to the same spatial and temporal assemblage. Second, desert tenebrionids are detritivorous and opportunistic animals [18,25], which therefore use the same basic resources. Third, desert tenebrionids are phylogenetically bounded because they belong to the same monophyletic group [26,27].

In arid ecosystems, sparse tufts of shrubs represent the primary driver of variation of many environmental variables, such as microclimate, soil water and nutrient availability [28,29,30,31,32,33,34], and many studies highlighted their paramount importance in determining the distribution and structure of ground-dwelling invertebrate communities [35,36,37,38,39]. However, these studies involved species that used different types of resources (e.g., including detritivores, herbivores, and predators) and which were phylogenetically unrelated. Moreover, these studies used pitfall traps operating for several days, which leads to the collection of species that are active in different parts of the day and in different days. Thus, collected species were not necessarily active at the same time. Therefore, these studies dealt with communities, not guilds or ensembles.

Gathering information at the ensemble level can be extremely useful, because it can shed light on the ecological role of shrubs and their species identity in structuring the distribution and assembly of ground-dwelling insect communities. This is essential for developing suitable management strategies for biodiversity conservation within arid ecosystems [40,41,42]. To the best of our knowledge, no study has previously investigated ground-dwelling insect ensembles in desert ecosystem. In this study, we investigated if the tenebrionid beetles inhabiting a desert ecosystem form different ensembles associated with different dominant vegetation types. In particular, we tested if: (1) different ensembles follow different patterns of dominance; (2) there was some association between tenebrionid species and plant species; (3) different ensembles exhibit different levels of diversity.

## 2. Materials and Methods

### 2.1. Study Area and Data Collection

The study was conducted in the Ulan Buh Desert. The Ulan Buh Desert is part of the Ala Shan Desert, which is the southernmost portion of the Gobi Desert, in the southwestern portion of Inner Mongolia (north-central China). The Ulan Buh Desert covers about 11,000 km^2^ and is surrounded by the Yellow River (east), the Langshan Mountains (north), the Bayan Urals Mountains (west), and the Helan Mountains (south).

The Ulan Buh Desert is part of the Hetao basin, a Cenozoic fault basin surrounded by the Ordos Plateau, the Helan Mountains and the Yinshan Mountains [43]. It is comprised of (1) a high-dune zone in the south (with ~100 m high pyramid dunes and compound mega dunes); (2) a low-dune zone in the west (dominated by ~10 m high fixed and semi-fixed dunes); (3) a dry wetland and lake-bed zone dominated by Quaternary lacustrine sediments in the north; and (4) a sand zone, characterized by low linear dunes near the mountains and by relatively high pyramid dunes further away from the mountains, in the east [44]. The Ulan Buh Desert is located at the margin of the Asian summer monsoon, on the transition zone between arid and semi-arid China, and is therefore characterized by a continental climate [44]. The climate in winter is mainly influenced by the dry, cold north-westerly winter monsoon that generates frequent dust storms, whereas the summer climate is dominated by the warm, moist south-easterly summer monsoon, which is responsible for most of the annual precipitation and rainstorms [45]. The Ulan Buh Desert is not the largest desert in Inner Mongolia, but it is considered to be of paramount socio-economic importance because of the presence of urban areas, agricultural fields, and factories. For this, various measures have been adopted to facilitate human presence, including the construction of roads, irrigation channels, sand barriers to fix the drifting sand, and the plantation of shrubs, trees and grass, which anchor sand and bring economic benefits to local farmers. A complete forest belt has been realized at the edge of the Ulan Buh Desert along the Yellow River.

**Figure 1:**
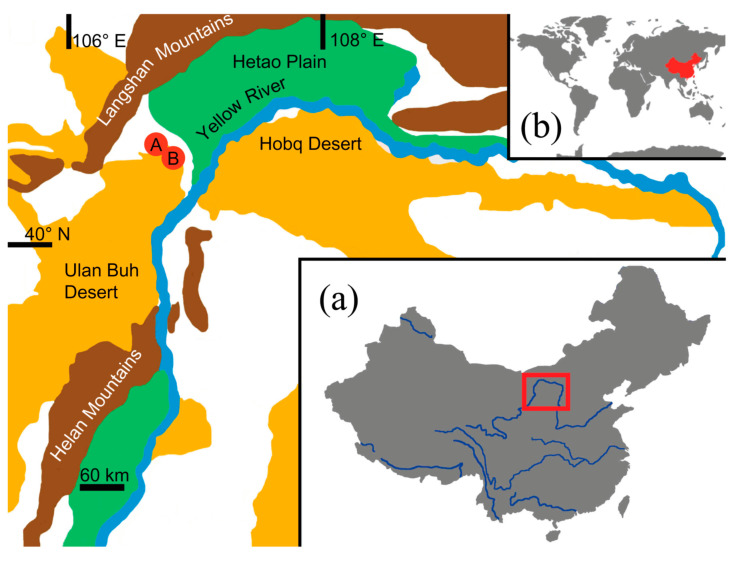
Schematic representation of the study area and location of the two sampling sites, A and B. The inset (**a**) shows the location of the study area in China, and the inset (**b**) the location of China.

The present study was conducted in the eastern sector of the Ulan Buh Desert (Figure 1). In this sector, the climate is characterized by a mean annual precipitation of 152.7 mm and a mean potential evaporation of 2351 mm, over 15 times more than the mean annual precipitation [46]. The mean annual air temperature is 7.5 °C, with a maximum of 38.7 °C in July and a minimum of −32.8 °C in January. The water for plant growth is mainly from seepages of upper diluvial fans. Such severe environmental conditions cause poor plant diversity and simple vegetation types represented mainly by desert scrubs, including *Artemisia ordosica* Krascheninnikov, *Artemisia sphaerocephala* Krascheninnikov, *Haloxylon ammodendron* (C. A. Meyer) Bunge, *Nitraria tangutorum* Bobrov and *Psammochloa villosa* (Trinius) Bor. Most of the vegetation is restricted to the lower part of the sand dunes and in the interdunes, with a coverage varying from 10% to 30% [46].

**Figure 2:**
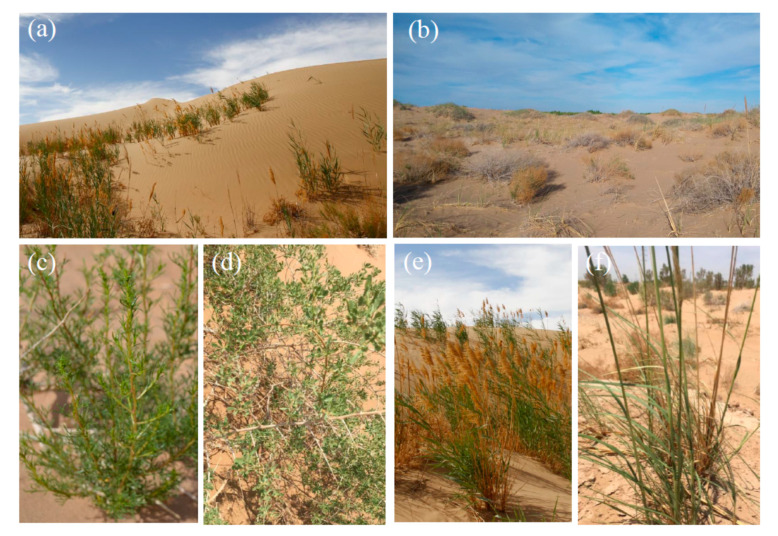
Sampling sites and vegetation in the Ulan Buh Desert (Gobi Desert, Inner Mongolia). (**a**) Site A; (**b**) Site B; (**c**) *Artemisia sphaerocephala*; (**d**) *Nitraria tangutorum*; (**e**); *Phragmites australis*; (**f**) *Psammochloa villosa*.

Sampling was done between 19 and 20 June 2018 in two sites: one (Site A) located at 40°26.501′ N and 106°34.718′ E (1035 m elevation) and the other (Site B) located at 40°25.010′ N and 106°39.841′ E (1042 m elevation) (Figure 1). Vegetation in Site A was mainly represented by the following three plant species: (1) *Artemisa sphaerocephala*, a typical desert species, characterized by strong, long and shortly branched woody stocks, largely verdant and dominant during our sampling; (2) *Nitraria tangutorum*, a typical plant of sandy areas, which forms tall, prostrate, spiny and much branched shrubs; during our sampling, most of the shrubs were already dry and bore fruits; (3) *Phragmites australis* (Cavanilles), a reed with extensive creeping rhizomes and very high culms (up to two m); since this species forms large colonies in moist places along river banks and lake margins, its abundance in this site suggests the presence of humid, albeit localized conditions (Figure 2). Vegetation in Site B was mainly represented by *Ar. sphaerocephala*, *N. tangutorum*, and *Psammochloa villosa*, a grass superficially resembling *Ph.- australis*, but typical of sand dunes (Figure 2). Vegetation was very sparse in Site A, and relatively dense in Site B.

Beetles were collected by means of pitfall traps. At each site, for each dominant plant species, we selected at random three tufts. Beneath each tuft of the same plant species, a pitfall trap with a different fluid was placed. Pitfall traps were made of plastic cups (diameter: 9 cm, depth: 10 cm) dug into the ground and filled with 50 mL of different fluids to test their possible different influence in beetle trapping: (1) propylene glycol, (2) beer and (3) a mixture of beer and propylene glycol (3:1). Thus, three traps per plant species were used, making a total of 9 traps per site. Tufts of the same species were at a distance of at least 50 m, and tufts of different species were at least 250 m apart. Tuft size varied depending on the species and its cover. For this reason, we made it so that the area between tufts was virtually void of plants. Pitfall traps were put down in the afternoon (at 5–6 P.M.) and emptied after 16 h (at 9–10 A.M.) to collect beetles mostly active from sunset to the dawn. No lid was used. This sampling strategy was used to assure that local beetle populations were not over-captured due to an unnecessary sampling effort [39]. Previous studies indicated that the use of a few traps operating for a short period is adequate to sample tenebrionids in this type of habitat [22,23,47,48].

We used a Spearman rank correlation coefficient (*r_s_*) to investigate the possible influence of bait composition on tenebrionid species abundance distributions (SADs). Because SADs obtained from the three fluids for each plant species in both sites were strongly correlated (0.9 < *r_s_* < 1, 0.001 = *P* < 0.0001), we pooled the data from the three pitfall traps placed beneath the same plant species. Trap content was preserved in jars filled with 95% ethanol and sorted in the laboratory. Tenebrionids were identified to species level. All material is preserved in the Museum of Hebei University.

### 2.2. Data Analysis

Insect abundances in harsh environments and early successional stages are known to follow the so-called “niche pre-emption” model, in which the sizes of the niche hypervolumes (measured by species relative abundances) are sequentially pre-empted by the most abundant to the least abundant species [49,50,51]. In this model, the first species in the sequence occupies a fraction *k* of resource hypervolume, the second species a fraction *k* of the hypervolume not occupied by the first, and so on. This model is mathematically expressed by the geometric series. To test if species abundance distributions matched the geometric series, we modelled rank–abundance curves [52,53,54] with the Ordinary Least Squares (OLS) regression approach described by Fattorini [50]. This approach is based on the fact that, if species are ranked from the most to the least abundant, and abundances are logarithmically transformed, the geometric series is linearized and can be therefore fitted using an OLS regression. Thus, the coefficient of determination *R*^2^ can be used as a goodness-of-fit measure, and differences between slopes can be assessed by analysis of covariance (ANCOVA). The niche pre-emption parameter *k* was calculated following He and Tang [55].

To investigate if there was some association between tenebrionid species and plant species (i.e., if tenebrionid species occurred with different proportions beneath the three plant species), we applied a χ^2^ test to a species × per plant contingency table. After this overall χ^2^ test, we used single species χ^2^ tests to assess if tenebrionid abundances beneath the three plant species deviated significantly from a uniform distribution. This analysis was restricted to species with expected frequencies >5.

Variations in ensemble structure among the tenebrionids associated with the three dominant plant species were investigated by using diversity indices. Among the vast multitude of indices proposed to express diversity, Hill numbers *^q^D*(∞) have been increasingly used because they seem to represent an intuitive and statistically rigorous alternative to other diversity indices [56]. Hill numbers combine information on species richness, species rarity and species dominance, and they are all expressed in the same units (i.e., effective number of species), being therefore comparable between each other. In Hill numbers, the diversity order *q* determines the measure’s sensitivity to species relative abundances. Hill numbers include three widely used species diversity measures as special cases: species richness (*q* = 0), exponential Shannon diversity (*q* = 1) and Simpson diversity (*q* = 2). Thus, for each ensemble, we calculated a diversity profile curve, which plots Hill numbers *^q^D*(∞) as a function of order *q*, 0 ≤ *q* ≤ 4. Calculations were done using PAST 3.25 [57].

## 3. Results

**Figure 3:**
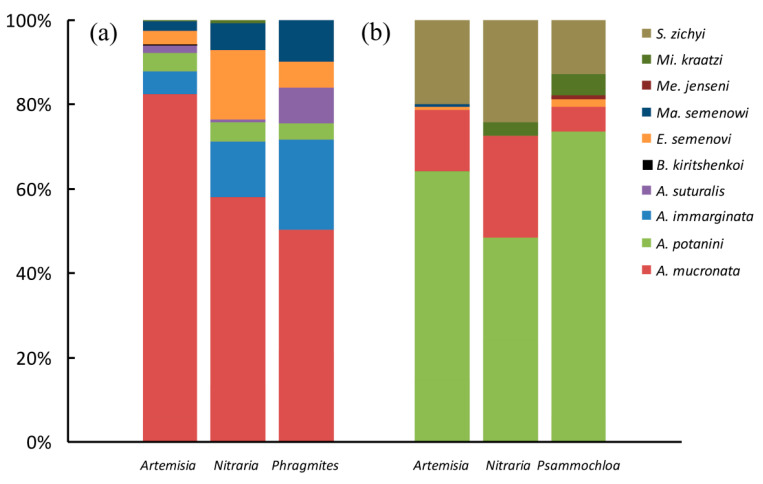
Proportion of the tenebrionid beetle species collected beneath four plant species in two sites (A and B, panels a and b, respectively) in the Ulan Buh Desert (Gobi Desert, Inner Mongolia, China). For insect species names, see Table 1.

**Figure 4:**
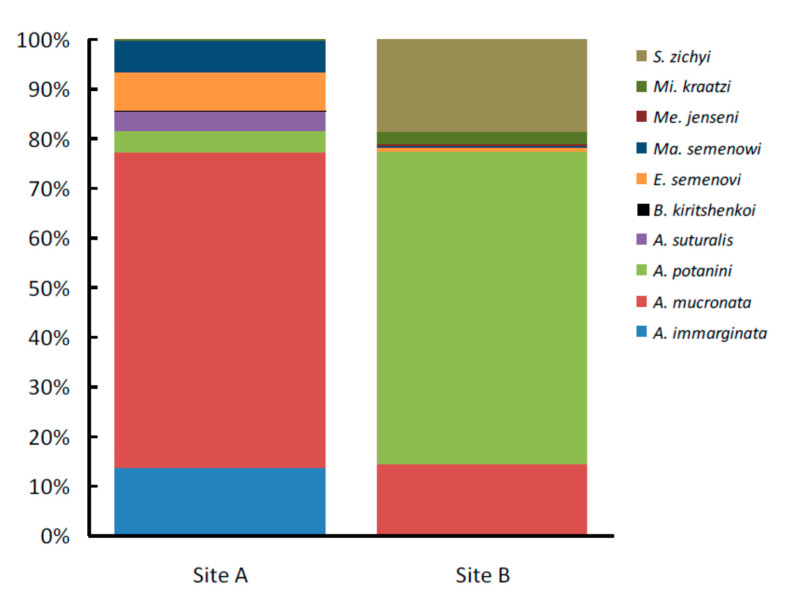
Proportion of the tenebrionid beetle species collected in two sites (A and B) in the Ulan Buh Desert (Gobi Desert, Inner Mongolia, China). For insect species names, see Table 1.

In total, we collected 1479 individuals belonging to 10 species (Table 1, Figure 3 and Figure 4). In both sites, the total tenebrionid beetle abundance varied significantly between the three ensembles (χ^2^ = 26.493, df = 2, *P* < 0.0001 in Site A, and χ^2^ = 15.131, df = 2, *P* < 0.0001 in Site B, respectively). Moreover, the total number of beetles trapped in Site A was three times that of Site B (χ^2^ = 391.560, df = 2, *P* < 0.0001). All ensembles were characterized by a strong dominance of certain species. In particular, *A. mucronata*, which represented about 64% of the total number of tenebrionid beetles collected during our sampling in Site A, accounted for 82% of tenebrionids beneath *Ar. sphaerocephala*, 58% of tenebrionids beneath *N. tangutorum*, and 50% of tenebrionids beneath *Ph. australis*, respectively. In this site, the second most abundant species, *A. immarginata*, represented about 14% of the collected individuals (6% beneath *Ar. sphaerocephala*, 13% beneath *N. tangutorum*, and 21% beneath *Ph. australis*, respectively). Thus, the two most abundant species, taken together, represented more than 77% of the total number of individuals. In Site B, *A. potanini* represented more than 63% of the collected tenebrionid beetles, accounting for 64% of tenebrionids beneath *Ar. sphaerocephala*, 48% of tenebrionids beneath *N. tangutorum*, and 74% of tenebrionids beneath *P. villosa*, respectively. In this site, the second most abundant species, *S. zichyi*, represented about 19% of the collected individuals (20% beneath *Ar. sphaerocephala*, 24% beneath *N. tangutorum*, and 13% beneath *P. villosa*, respectively). Thus, the two most abundant species, taken together, represented about 82% of the total number of tenebrionids in this site.

**Figure 5:**
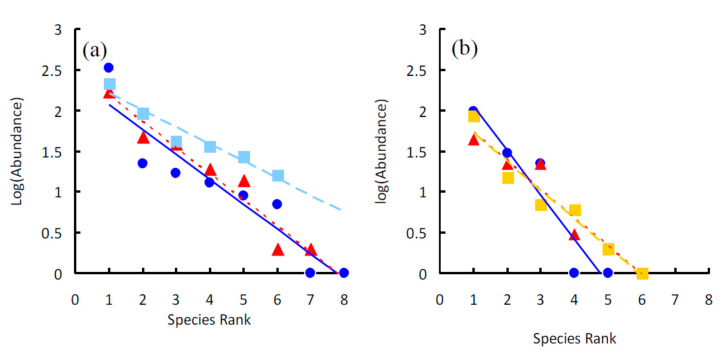
Rank–abundance plots of the tenebrionid beetles collected beneath four plant species in two sites in the Ulan Buh Desert (Gobi Desert, Inner Mongolia, China): *Artemisia sphaerocephala* (blue dots, continuous line), *Nitraria tangutorum* (red triangles, dotted line), *Phragmites australis* (light blue squares, broken line) and *Psammochloa villosa* (yellow squares, broken line). Species are ranked from the most to the least abundant (*x*-axis). Species abundances are log-transformed (*y*-axis). The data were fitted using linear regressions. (**a**) Rank–abundance plots for the ensembles of Site A. (**b**) Rank–abundance plots for the ensembles of Site B.

**Figure 6:**
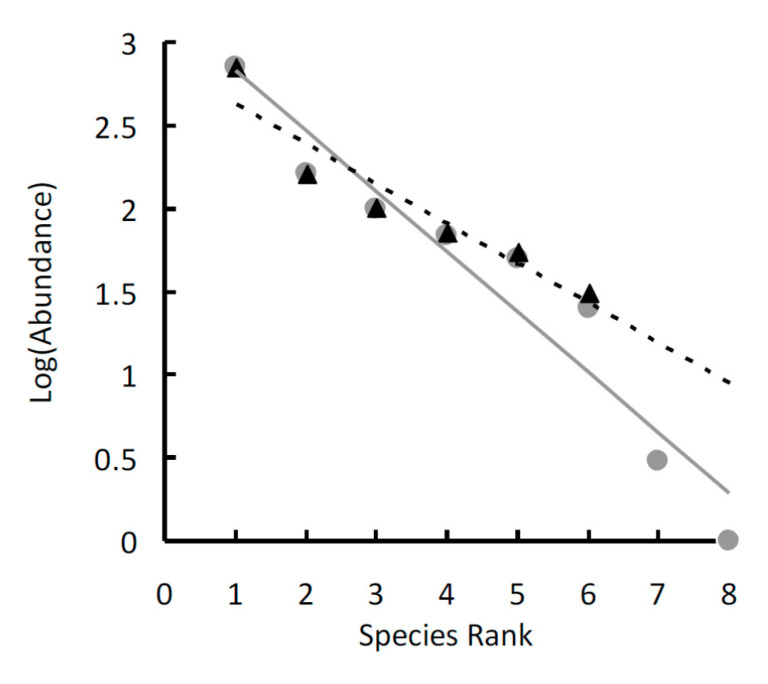
Rank–abundance plots of the tenebrionid beetles collected in two sites in the Ulan Buh Desert (Gobi Desert, Inner Mongolia, China): Site A (gray dots, continuous line), and Site B (black triangles, dotted line). Species are ranked from the most to the least abundant (*x*-axis). Species abundances are log-transformed (*y*-axis). The data were fitted using linear regressions.

The goodness-of-fit statistics of the OLS regression lines (Table 2) indicated that the geometric series fitted adequately all species ensembles (Figure 5) and the two whole communities (Figure 6). The lack of significance of the regression line for *N. tangutorum* in Site B is due to the small number of involved species (only four data points), whereas the goodness-of-fit is high (more than 80% of variance explained).

In both sites, the slopes of the three regression lines were not significantly different (ANCOVA for the homogeneity of slopes: *F*_2,15_ = 1.385, *P* = 0.281 for Site A, and *F*_2,9_ = 1.880, *P* = 0.208 for Site B, respectively). No difference was found between the two sites (*F*_1,12_ = 3.371, *P* = 0.096). In Site A, *k* was higher (about 0.56) beneath *Ar. sphaerocephala*, slightly lower beneath *N. tangutorum* (*k* = 0.52) and much lower (*k* = 0.34) beneath *Ph. australis*. In Site B, *k* was highest beneath *Ar. sphaerocephala* (*k* = 0.68), and lower beneath *N. tangutorum* and *P. villosa*, (attaining a value of about 0.59 in both cases). *k* values for the two whole communities were similar (*k* = 0.61 in both cases).

We found an association between species abundances and plant species in both sites (χ^2^ = 170.925, df = 14, *P* < 0.0001 for Site A, and χ^2^ = 33.401, df = 12, *P* = 0.001 for Site B, respectively), which indicates that species occur with different proportions in the three sampled plants (Figure 3).

The separate χ^2^-tests for uniform distributions in Site A revealed that *A. mucronata* (χ^2^ = 55.762, df = 2, *P* < 0.0001), *A. immarginata* (χ^2^ = 52.275, df = 2, *P* < 0.0001), *A. suturalis* (χ^2^ = 44.933, df = 2, *P* < 0.0001), *E. semenovi* (χ^2^ = 21.159, df = 2, *P* = 0.0003) and *M. semenowi* (χ^2^ = 24.543, df = 2, *P* < 0.0001) occurred with different abundances in the three ensembles, whereas *A. pontanini* had a uniform distribution (χ^2^ = 0.298, df = 2, *P* = 0.862) (Table 1).

In Site B, both *A. mucronata* (χ^2^ = 8.824, df = 2, *P* = 0.012), and *A. pontanini* (χ^2^ = 20.678, df = 2, *P* < 0.0001) had different abundances in the three ensembles, whereas no significant difference was observed for *S. zichyi* (χ^2^ = 5.045, df = 2, *P* = 0.803) (Table 1). Namely, *A. mucronata* was mostly associated with *Ar. sphaerocephala* (in Site A) and *N. tangutorum* (in Site B). *A. pontanini* in Site B was mostly associated with *Ar. sphaerocephala* and *P. villosa*, being scarce beneath *N. tangutorum*. In Site A, *A. immarginata, A. suturalis* and *M. semenowi* were mostly associated with *Ph. australis*, whereas *E. semenovi* was mostly associated with *N. tangutorum.*

**Figure 7:**
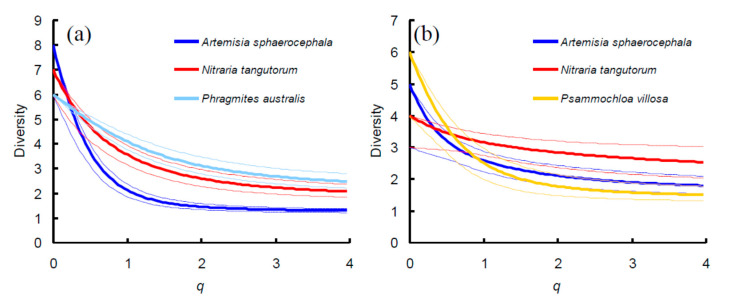
Diversity profile curves for the tenebrionid beetles collected beneath four plant species in two sites (A and B) in the Ulan Buh Desert (Gobi Desert, Inner Mongolia, China): *Artemisia sphaerocephala* (blue line), *Nitraria tangutorum* (red line), *Phragmites australis* (light blue line), and *Psammochloa villosa* (yellow line). Thin lines are 95% confidence intervals based on 2000 replicates. (**a**) Diversity profiles for the ensembles of Site A. (**b**) Diversity profiles for the ensembles of Site B.

In Site A, the three ensembles showed similar values of diversity for *q* = 0 (which corresponds to species richness), but differences among ensembles became clear at increasing *q*, showing that diversity was lowest in the *Ar. sphaerocephala* ensemble and higher in the *N. tangutorum* and *Ph. australis* ensemble (Figure 7a). In Site B, the three ensembles showed similar values of diversity for *q* = 0, but, again, differences among ensembles became clear at increasing *q* (Figure 7b), although the increase in difference among ensembles with increasing *q* appears less clear here. At *q* =1 (corresponding to the exponential Shannon Weaner index of diversity), *Ar. sphaerocephala* and *P. villosa* ensembles had virtually identical diversity values, but the *N. tangutorum* ensemble attained a higher value, although confidence intervals are broadly overlapped. A further increase in *q* determines a better separation between the three ensembles, with diversity being highest in the *N. tangutorum* ensemble, lowest in the *P. villosa* ensemble and intermediate in the *Ar. sphaerocephala* ensemble.

**Figure 8:**
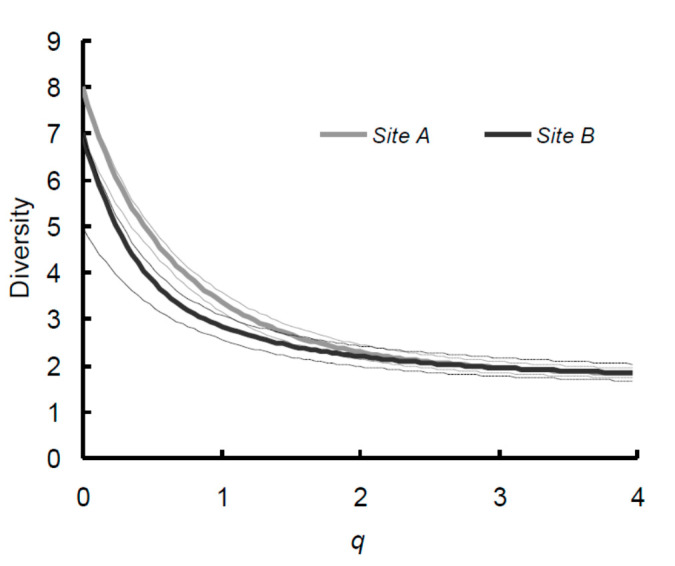
Diversity profile curves for the tenebrionid beetles collected in two sites (A and B) in the Ulan Buh Desert (Gobi Desert, Inner Mongolia, China): Site A (gray) and Site B (black). Dotted lines are 95% confidence intervals based on 2000 replicates.

Diversity curves calculated for the whole communities in the two sites show an increasing similarity at increasing values of *q*, with, however, Site A showing a higher diversity for the values corresponding to richness and Simpson diversity (Figure 8).

## 4. Discussion

Tenebrionids living in Central Asian arid ecosystems form relatively simple communities composed of few species, typically between one and 12 [22,23,24,47,48]. Thus, the total species richness recorded in our study (eight species in Site A, and seven species in Site B, respectively) falls within the range observed from other sites with similar environmental conditions. In particular, it is interesting to compare our results with studies conducted with similar short-time sampling periods. In a study on the beetle communities in the Altai Mountains (Mongolia), Khurelpurev and Pfeiffer [48] used 10 traps per site that were examined twice a day (at 9:00 A.M. and 9:00 P.M.), which implies a number of hours greater than ours. The highest values of species richness and abundance in Khurelpurev and Pfeiffer’s study [48] were recorded in a remote steppe site in the Gobi-Altai province, where they collected 64 individuals belonging to 10 species. Their sampling in two true desert areas led to the collection of 11 individuals belonging to six species, and 13 individuals belonging to five species, respectively.

In another broad scale study on beetle communities in different ecosystems in Mongolia, Pfeiffer and Bayannasan [47] used a much more intense sampling protocol, with 75 traps (baited Petri dishes) per site, active at least for a full cycle of diurnal and nocturnal surface temperatures, thus for a longer time than in our study. In Pfeiffer and Bayannasan’s study [47], forest steppe, steppe, semi desert and desert ecosystems were sampled. The desert areas were represented by four sites in the Gobi-Altai, East Gobi and Transaltai-Gobi areas. The number of tenebrionids collected from these desert sites varied between 12 and 129 and species richness between three and eight species, respectively. Although using fewer traps per site, we collected much more individuals (two or three orders of magnitude), but a similar number of species.

Moreover, our number of species is consistent with those recorded in similar environments using much more extensive sampling. Li et al. [24] conducted a study in an artificial oasis in the western Gansu Province, northwestern China. They used 360 pitfall traps, for one week in May and one week in August. This led to the collection of 9670 individuals belonging to nine species. In a desert site close to this oasis, Liu et al. [22] conducted a study with 48 traps in three sampling periods (in May, July and September), each consisting of 15 consecutive days. They collected 6248 individual tenebrionids belonging to seven species. Finally, in a study conducted in a sandy desert ecosystem in the middle reaches of the Heihe River (northwestern China), Liu et al. [23] used 36 traps for three sampling periods (in May, July and September), each consisting of 15 consecutive days. They collected 8707 tenebrionids belonging to seven species. Thus, these results suggest that our sampling approach was adequate to collect virtually all species present in the study sites, despite the small number of traps used and the short period of sampling.

The tenebrionid communities inhabiting desert ecosystems are not only composed of few species, but also show a simple structure characterized by a high dominance of a few species with large numbers of individuals [16,58,59]. This is clearly illustrated, in our case, by the high dominance of *A. mucronata* and *A. immarginata*, which, taken together, represented about 80% of the total number of individuals collected in one sampling site, whereas *A. potanini* and *S. zichyi*, taken together, represented about 80% of the tenebrionids collected in the other sampling site.

This pattern of species dominance is clearly reflected by the good fit provided by the geometric series, and it is indicative of a community dominated by *r*-selected species. This is in contrast with findings concerning other environments, where most tenebrionids operate under a *K*-mode of selection [60], but it is consistent with the fact that tenebrionid species living in other arid environments, such as the Mediterranean sand dunes, are typically *r*-selected [25,61,62,63]. Both desert and beach-dune systems are strongly influenced by harsh climatic and edaphic factors, and, in both these environments, trophic food sources are scarce and rather homogeneous, being mostly represented by vegetable detritus. Thus, these environments can be best colonized by a reduced number of sand-specialized and *r*-selected tenebrionid species that can use decaying organic matter.

Insect species abundance distributions are known to follow a geometric series in the early stages of successions, where communities are mainly composed of small-sized, relatively short-lived, or opportunistic species with fluctuating populations [49]. More generally, the geometric series has been widely used to describe communities of early successions [64,65], which are subject to disturbances [66,67,68] or that occupy poor habitats [69,70]. Both desert and beach dunes recapitulate all these characteristics because they can be viewed as early succession stages subject to strong disturbances and with a very low productivity, therefore being perfectly qualified to test the validity of the geometric series model.

The observed values of the slopes of the regression lines obtained in this study (from −0.55 to −0.21) are, however, higher than those known from tenebrionids communities of Mediterranean beach dune systems (which are comprised between −0.29 and −0.25; [25,63]). As regards the value of *k*, which indicates the sequential, constant proportion of the total number of individuals in the community, the *Ar. sphaerocephala* ensemble was that with the highest *k* in both sites. In Site A, the *k* of the *Ar. sphaerocephala* ensemble was about 0.56, which means that the most common species would represent about 56% of individuals in the ensemble, the second most common species would represent half of the remaining half (22%), the third, half of the remaining quarter (11%), and so on. This indicates a rather sharp decline in the species niche hypervolumes, as expected for an assemblage of beetles inhabiting harsh environments. *k* was even higher in the *Ar. sphaerocephala* ensemble in Site B (*k* = 0.68). In general, Site B was characterized by higher *k*-values than Site A, which indicates a stronger dominance effect. This might be a reflection of a higher productivity of Site B, which has a denser plant cover. Overall, these *k* values are higher than those observed in tenebrionid communities of Mediterranean dune ecosystems (*k* = 0.43–0.49; [62,63]) and close to the highest recorded from other sites in the Gobi Desert (*k* = 0.21–0.64) [71].

The diversity profiles indicate that, in both sites, the distinction between ensembles increases at increasing values of *q*, and becomes clear when *q* = 2, which corresponds to the Simpson diversity index. This index is, in turn, the reciprocal of Simpson dominance, which represents the probability that two randomly chosen individuals belong to the same species (see [72] for details). More generally, the slope of the diversity curves reflects the unevenness of species relative abundances. The more uneven the distribution of relative abundances, the more steeply the curve declines, whereas, for completely even relative abundances, the curve is a constant at the level of species richness [56]. In Site A, the *N. tangutorum* curve had a pattern similar to that of *Ph. australis*, with broadly overlapping confidence intervals; by contrast, in Site B, the *N. tangutorum* curve showed the less steep decline.

Both *Ar. sphaerocephala* and *N. tangutorum* have a similar, perennial shrubby habit that can produce more shadow and maintain more moisture than *Psammochloa*, which has a grass, non-branched habit and cannot make an umbrella-like canopy, making the underlying soil more exposed to sun, wind and sand action. These characteristics may contribute to make these plants more attractive to tenebrionids.

**Figure 9:**
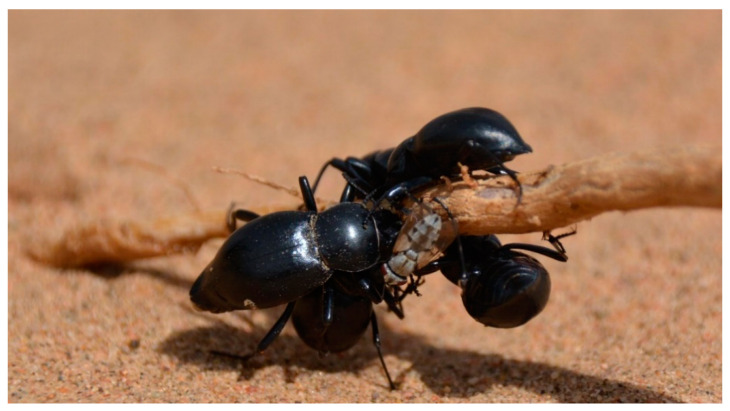
*Anatolica mucronata* feeding on damaged tissues of *Artemisia sphaerocephala*.

**Figure 10:**
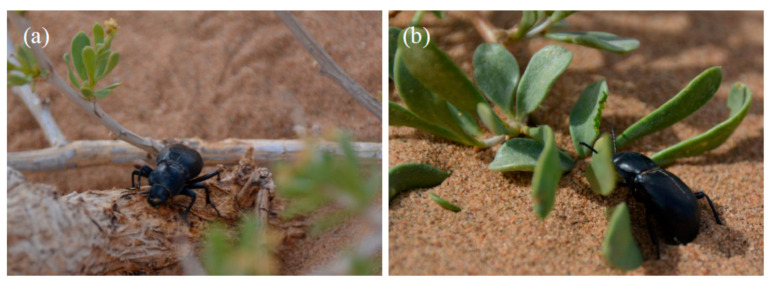
*Anatolica potanini* feeding on *Nitraria tangutorum*. (**a**) Feeding on damaged tissues; (**b**) feeding on leaves.

Moreover, these plants may provide tenebrionids with water and nutrients from their damaged tissues. Desert shrubs such as *N. tangutorum* and *Ar. sphaerocephala* can reproduce sexually or asexually, and the harsh conditions of the environment lead the plants to create branch-derived clonal offshoots. In these plants, the sand-covered stems develop adventitious roots downwards, which generate new shoots upwards and can then generate a new whole ramet (member of a clonal ensemble). Desert plants are, however, exposed to an almost continuous disturbance due the aeolian sand activity, which causes an erosion of plant tissues. Plants on the windward side commonly lose water and sap due to the wind erosion exposing stem and root tissues, making droplets of their fluids available to the beetles and other invertebrates that lack specialized sucking mouthparts. Thus, damaged plant parts of *N. tangutorum* and *Ar. sphaerocephala* may represent an important source of water and nutrients for tenebrionid beetles (Figure 9 and Figure 10a).

Moreover, *N. tangutorum* has a more succulent leaf than *Ar. sphaerocephala* (Figure 10b). The higher water and salt contents in *N. tangutorum* leafs, compared to *Ar. sphaerocephala*, could further enhance the ability of this plant of sustaining a more diverse ensemble when vegetation is extremely scarce (Figure 7b).

In Site A, *P. villosa* is replaced by another grass, *Ph. australis*. Despite the general shape of *Ph. australis* is very similar to that of *P. villosa*, the tenebrionid ensemble recorded beneath *Ph. australis* was characterized by a high diversity, similar to that of *N. tangutorum*. This might be explained by the fact that *Ph. australis* grows in humid places, which can be attractive to tenebrionid because of the higher moisture. This can also explain the overall high diversity in Site A compared to Site B.

It has been demonstrated that biotopes separated by less than 10 km, but with strong differences in soil characteristics, host tenebrionid communities with a similar species composition but a very different community structure, and a local selection mechanism has been invoked, suggesting that soil characteristics regulated species abundances [21]. In the present study, we found that in the two investigated sites, which are less than eight km apart but have similar environmental conditions, the most dominant species is not the same, but the ensemble organization is very similar. This indicates that similar environmental conditions lead to similar insect ensemble organization, independent of species identity. At the same time, our results open a new question: why is the most abundant species not the same? In other words, why is *A. mucronata* the most abundant species in Site A, whereas *A. potanini* is the most abundant in Site B? Further research is needed to assess if this is due to ecological factors that favor a certain species in a site and another one in the other, to competition or to random factors (i.e., the species that first occupies a certain area increases rapidly and prevents the other to being abundant). For example, it is interesting that *S. zychi*, which is 17–20 mm long [73], and which is present in Site B but not in Site A, might be a competitor of the large *Anatolica* species, such as *A. immarginata* (18–22 mm), *A. mucronata* (13–16 mm) and *A. suturalis* (15 mm) (measures taken from [74]). Thus, we can hypothesize that the presence of *S. zychi* in Site B might be the reason for the scarcity or the complete absence of these large *Anatolica* species, which, in contrast, are dominant in Site A. In turn, the low number of larger *Anatolica* in Site B might have favored the dominance of the smaller *A. potanini* (9–14 mm) in this site. Since tenebrionid communities seem to be poorly influenced by competition [62], further, ad hoc research is needed to test this hypothesis.

In general, Site A has a lower plant cover, and more collected beetles. This might be counterintuitive if tenebrionid abundance were enhanced by productivity, which is expected to be higher in Site B, because of the positive relationship between vegetation cover and productivity [75]. However, in our specific case, this might be explained by assuming that a more scattered plant distribution could force beetles to concentrate them, and hence to increase their density, near the sparse plants in the less vegetated site, whereas, in the more vegetated site, beetles were more dispersed and hence assumed a lower density, a pattern already observed in the tenebrionids inhabiting coastal dunes [76,77].

We also found, within each site, that tenebrionid species were differently associated with different plant species, showing some habitat selection. Thus, the same basic tenebrionid community (i.e., the assembly at site level) can lead to different ensemble organizations in association with different dominant vegetation. Thus, a filtering process may operate at a very small spatial scale, leading to differences in species proportions (and diversity parameters) even among very close biotopes (i.e., the three dominant plants). Although some species were too rare to assess their plant preference, our data support that, at least some are distributed with a different abundance beneath the four plant species. These differences might be related to different preferences for different characteristics in plant cover, vegetation complexity and litter production, which requires further investigation.

We found that the same species has different preferences for the four investigated plants according to the site. This can be explained by different reasons. First, differences in the overall abundances at the site level can be important. For example, the fact that *A. mucronata* is mostly associated with *Ar. sphaerocephala* in Site A, but with *N. tangutorum* in Site B, can be due to the fact that this species is the most abundant in Site A, whereas the most abundant species in Site B is *A. potanini*. Thus, factors influencing overall abundance at site level may have consequences at finer scales. Second, there may be ecological factors that change tenebrionid preferences; thus, the same plant can be favored in a certain site, but not in another one. Third, there may be random factors, i.e., the species that, in a given site, first occupies a certain plant, increases rapidly and prevents others from being abundant there. In the future, testing these hypotheses with a broader spatial scale would give important insights into our understanding of tenebrionid ensemble organization.

We conducted our sampling in the beginning of summer, when tenebrionid beetles have high values of activity [23]. Thus, our snapshot sampling protocol produced a reliable picture of the tenebrionid ensembles during the maximum activity period of tenebrionid species but does not take into account temporal variations in ensemble structure. For example, *Platyope ordossica* Semenow, 1907, is very common in this desert, but it appears in large numbers in early May (G.R. personal observations), and was completely absent in our sampling, which was conducted at the end of June. More generally, species activity patterns can change through seasons, which will generate ensembles with different characteristics. In the future, it would be interesting to see if the patterns highlighted in this study vary across seasons.

## 5. Conclusions

We found that tenebrionids form different ensembles associated with the different dominant plant species. At the same time, all these ensembles are characterized by similar patterns of dominance, with a high dominance of few species with large numbers of individuals, following the “niche pre-emption” model, and a steep decline in the diversity profiles. This indicates that similar environmental conditions lead to similar insect ensemble organization, although the most abundant species vary, which suggests a role for microhabitat selection. Further research, increasing the spatio-temporal sampling effort, will be useful to test this hypothesis.

## Figures and Tables

**Table 1:** Number of collected tenebrionid beetles beneath tufts of four plant species in two sites in the Ulan Buh Desert (Gobi Desert, Inner Mongolia). Plant names: Ar. sph. = *Artemisia sphaerocephala*; N. tan. = *Nitraria tangutorum*; Ph. aus. = *Phragmites australis*; P. vil. = *Psammochloa villosa.* Asterisks (*) indicate that species abundance across the three ensembles deviated significantly from a uniform distribution (see text for details).

Tenebrionid Species	Ar. sph.	N. tan.	Ph. aus.	Total A	Ar. sph.	N. tan.	P. vil.	Total B
*Anatolica mucronata* Reitter, 1889	328	170	215	713 *	22	22	7	51 *
*Anatolica potanini* Reitter, 1889	17	14	16	47	97	44	86	227 *
*Anatolica immarginata* Reitter, 1889	22	39	92	153 *	0	0	0	0
*Anatolica suturalis* Reitter, 1889	7	2	36	45 *	0	0	0	0
*Microdera kraatzi alashanica* Skopin, 1964	1	2	0	3	0	3	6	9
*Epitrichia semenovi* Bogachev, 1949	13	48	27	88 *	1	0	2	3
*Sternotrigon zichyi* (Csiki, 1901)	0	0	0	0	30	22	15	67
*Mantichorula semenowi* Reitter, 1889	9	19	42	70 *	1	0	0	1
*Melanesthes jenseni meridionalis* Kaszab, 1968	0	0	0	0	0	0	1	1
*Blaps kiritshenkoi* Semenov et Bogatshev, 1936	1	0	0	1	0	0	0	0
**Total**	398	294	428	1120	151	91	117	359

**Table 2:** Regression parameters (intercept and slope), goodness of fit (*R*^2^), t-value (*t*) and probability (*P*) of geometric series models for the tenebrionid ensembles associated with four plant species in two sites in the Ulan Buh Desert (Gobi Desert, Inner Mongolia). For each model, the niche pre-emption parameter *k* is also reported. Errors refer to standard errors.

Ensemble	Intercept	Slope	*R* ^2^	*t*	*P*	*k*
Site A						
*Artemisia sphaerocephala*	2.374 ± 0.245	−0.305 ± 0.049	0.868	−6.284	0.001	0.563
*Nitraria tangutorum*	2.503 ± 0.161	−0.321 ± 0.036	0.941	−8.943	<0.001	0.518
*Phragmites australis*	2.416 ± 0.104	−0.209 ± 0.027	0.938	−7.783	0.001	0.340
Total Site A	3.194 ± 0.214	−0.364 ± 0.042	0.925	−8.590	<0.001	0.609
Site B						
*Artemisia sphaerocephala*	2.597 ± 0.353	−0.545 ± 0.106	0.897	−5.119	0.014	0.681
*Nitraria tangutorum*	2.076 ± 0.333	−0.350 ± 0.122	0.806	−2.880	0.102	0.591
*Psammochloa villosa*	2.076 ± 0.166	−0.353 ± 0.043	0.945	−8.302	0.001	0.590
Total Site B	2.865 ± 0.157	−0.239 ± 0.040	0.899	−5.951	0.004	0.611
